# Clinical characteristics and complications of rotavirus gastroenteritis in children in east London: A retrospective case-control study

**DOI:** 10.1371/journal.pone.0194009

**Published:** 2018-03-22

**Authors:** Konstantinos Karampatsas, Leanne Osborne, May-Li Seah, Cheuk Y. W. Tong, Andrew J. Prendergast

**Affiliations:** 1 Department of Paediatrics, The Royal London Hospital, Barts Health NHS Trust, London, United Kingdom; 2 Department of Virology, The Royal London Hospital, Barts Health NHS Trust, London, United Kingdom; 3 Blizard Institute, Queen Mary University of London, London, United Kingdom; University of Hong Kong, HONG KONG

## Abstract

**Background:**

Rotavirus is the leading cause of acute gastroenteritis in children and is associated with neurological complications such as seizures and encephalopathy. The aim of this study was to investigate the presentation and complications of rotavirus compared to non-rotavirus gastroenteritis in UK children.

**Methods:**

This was a retrospective, case-control, hospital-based study conducted at three sites in east London, UK. Cases were children aged 1 month to 16 years diagnosed with acute gastroenteritis between 1 June 2011 and 31 December 2013, in whom stool virology investigations confirmed presence of rotavirus by PCR. They were matched by age, gender and month of presentation to controls with rotavirus-negative gastroenteritis.

**Results:**

Data were collected from 116 children (50 cases and 66 controls). Children with rotavirus gastroenteritis tended to present more frequently with metabolic acidosis (pH 7.30 vs 7.37, P = 0.011) and fever (74% versus 46%; P = 0.005) and were more likely to require hospitalisation compared to children with non-rotavirus gastroenteritis (93% versus 73%; P = 0.019). Neurological complications were the most common extra-intestinal manifestations, but did not differ significantly between children with rotavirus-positive gastroenteritis (RPG) and rotavirus-negative gastroenteritis (RNG) (24% versus 15%, respectively; P = 0.24). Encephalopathy occurred only in children with rotavirus infection (n = 3, 6%).

**Conclusion:**

Rotavirus causes longer and more severe disease compared to other viral pathogens. Seizures and milder neurological signs were surprisingly common and associated with multiple pathogens, but encephalopathy occurred only in children with rotavirus gastroenteritis. Rotavirus vaccination may reduce seizures and presentation to hospital, but vaccines against other pathogens causing gastroenteritis are required.

## Introduction

Rotavirus is the most common cause of severe gastroenteritis in children under the age of 5 years worldwide [1]. The cumulative annual burden of rotavirus-associated gastroenteritis globally is about 25 million outpatient visits, 2 million hospital admissions, and 180,000–450,000 deaths in children <5 years of age [2]. In the European Union, it is estimated that rotavirus causes each year around 3.6 million episodes of gastroenteritis, 700,000 clinic visits, 87,000 hospitalizations and 231 deaths in children <5 years of age [3]. Most infections are community-acquired, but rotavirus is also a major cause of nosocomial diarrhoea especially in infants <6 months of age [4]. There is accumulating evidence that rotavirus infection is not limited to the intestine [5]. Neurologic manifestations, including afebrile or febrile convulsions, meningoencephalitis, encephalopathy and cerebellitis appear to be the most common extra-intestinal complications [6].

Two oral, live attenuated rotavirus vaccines, a pentavalent bovine-human reassortment vaccine (RotaTeq [RV5], Merck and Company, Whitehouse Station, NJ) and a monovalent human vaccine (Rotarix [RV1], GlaxoSmithKline Biologicals, Rixensart, Belgium), have been available since 2006 and are recommended by the World Health Organization for routine immunisation of all infants [7]. The oral monovalent vaccine [RV1] was introduced to the national immunisation programme in England and Wales on 1 July 2013, as a 2-dose schedule for infants at 2 and 3 months of age. A systematic review of studies from eight countries reported a 49–89% reduction in hospital admissions due to rotavirus infection and 17–55% decrease in hospitalisation rates for gastroenteritis of any cause in children <5 years within two years after vaccine was introduced [8]. A recent vaccine efficacy study from the UK reported a 77% reduction in laboratory-confirmed rotavirus infections and a 26% reduction in all-cause acute gastroenteritis–associated hospitalizations in infants <1 year of age [9].

Although the burden of rotavirus gastroenteritis is well recognised, little information is available concerning rotavirus-associated complications in the UK. The aim of the present study was to investigate presentation and complications of rotavirus compared to non-rotavirus gastroenteritis in UK children.

## Methods

### Study design

This was a retrospective, matched case-control, hospital-based study conducted at three sites in east London, UK: The Royal London Hospital, Newham University Hospital, and Whipps Cross University Hospital.

Cases and controls were identified from the computerized virology laboratory database, by listing all children (age 1 month to 16 years) undergoing stool PCR testing between 1 June 2011 and 31 December 2013. The start date was when multiplex PCR was introduced for detection of gastroenteritis viruses at our sites; the end date was six months after rotavirus vaccination was introduced in the national UK schedule. Two triplex PCR were used in the diagnostic test, one targeting rotavirus and norovirus genotype 1 and 2; and one targeting adenovirus 40/41, sapovirus and astrovirus [10]. Cases were children diagnosed with acute gastroenteritis in whom rotavirus was detected by PCR; controls were children diagnosed with acute gastroenteritis in whom rotavirus was not detected by PCR. Acute gastroenteritis was defined following review of case notes as an episode of ≥3 loose stools or forceful vomiting occurring within a 24-hour period in the absence of a previously diagnosed chronic gastrointestinal disease that could imitate acute gastroenteritis [11]. No formal sample size calculation was performed before data collection. Controls were matched to cases by age, gender and month of presentation (in respective order of priority). Age of presentation was matched within a range of 3 months for patients <1 year of age, 6 months for patients between 1 and 2 years old and 12 months for patients >2 years old. Month of presentation was matched within a range of 3 months before or after the month of presentation. Infants <30 days old and children who had received any doses of rotavirus vaccine were excluded.

### Data collection

Anonymised data were extracted from patients’ medical notes and the computerised data system by a single investigator and entered into a database. Clinical features and complications were defined as follows

- Reporting of dehydration was based on the documentation of the clinician in the notes or the electronic data system. If there was no clinical impression documented, a retrospective review performed by the investigator based on the clinical criteria outlined in NICE guidelines [12]. The same tool was used for children >5 years of age.
- Seizures were classified based on the International League Against Epilepsy (ILAE) scheme [13].
- Encephalopathy was defined as depressed or altered level of consciousness, lethargy, or personality change lasting >24 hours based on the criteria of Brighton Collaboration Encephalomyelitis/ADEM Working Group [14].
- Recording of reduced consciousness level was based on the documentation of the clinician in the notes or the electronic data system. If there was no AVPU or Glasgow Coma Scale (GCS) assessment documented, a retrospective review performed by the investigator and the recording was based on the clinical criteria of the Brighton Collaboration Encephalomyelitis/ADEM Working Group for decreased or absent response to environment, defined as “decreased or absent response to loud noise or painful stimuli, and/or decreased or absent eye contact, and/or inconsistent or absent response to external stimuli and/or decreased arousability and/or seizure associated with loss of consciousness” [14].

### Statistical analysis

Statistical analysis was done with GraphPad Prism 7. Differences between groups were analysed using Fisher’s exact test (two-tailed) for categorical variables and the Student t-test or Mann-Whitney test for continuous variables, after checking for normality of distribution using the D'Agostino-Pearson and Shapiro-Wilks tests.

### Ethics approval and consent to participate

The clinical study was approved by the Joint Research Management Office of Barts Health NHS Trust, London, UK (Reference number 010202). The study was conducted in accordance with the Research Governance Framework for Health and Social Care (2005), the World Medical Association Declaration of Helsinki (1996), the Data Protection Act (1998) and the current applicable regulatory requirements. Because this was a retrospective review of clinical records, written informed consent from caregivers was not obtained. Research studies such as ours that utilise previously collected (in the course of normal clinical care), non-identifiable data do not require review by or approval of an NHS Research Ethics Committee, or a formal University REC.

## Results

Of the 153 children with positive stool rotavirus PCR identified through the laboratory information system, the records of 136 (89%) were available for review; 86 were subsequently excluded because they were investigated and treated in a primary care setting without having to attend the hospital emergency department and therefore no clinical information was available. 157 control subjects were matched to cases by age, gender and month of presentation. 145 sets of notes (92%) were available for review. 79 cases were subsequently excluded for the same reasons as above. The final analysis therefore comprised 116 patients: 50 children with rotavirus-positive gastroenteritis (RPG), and 66 children with rotavirus-negative gastroenteritis (RNG); the final ratio of case:control matching was 1:1.3.

**Fig 1:**
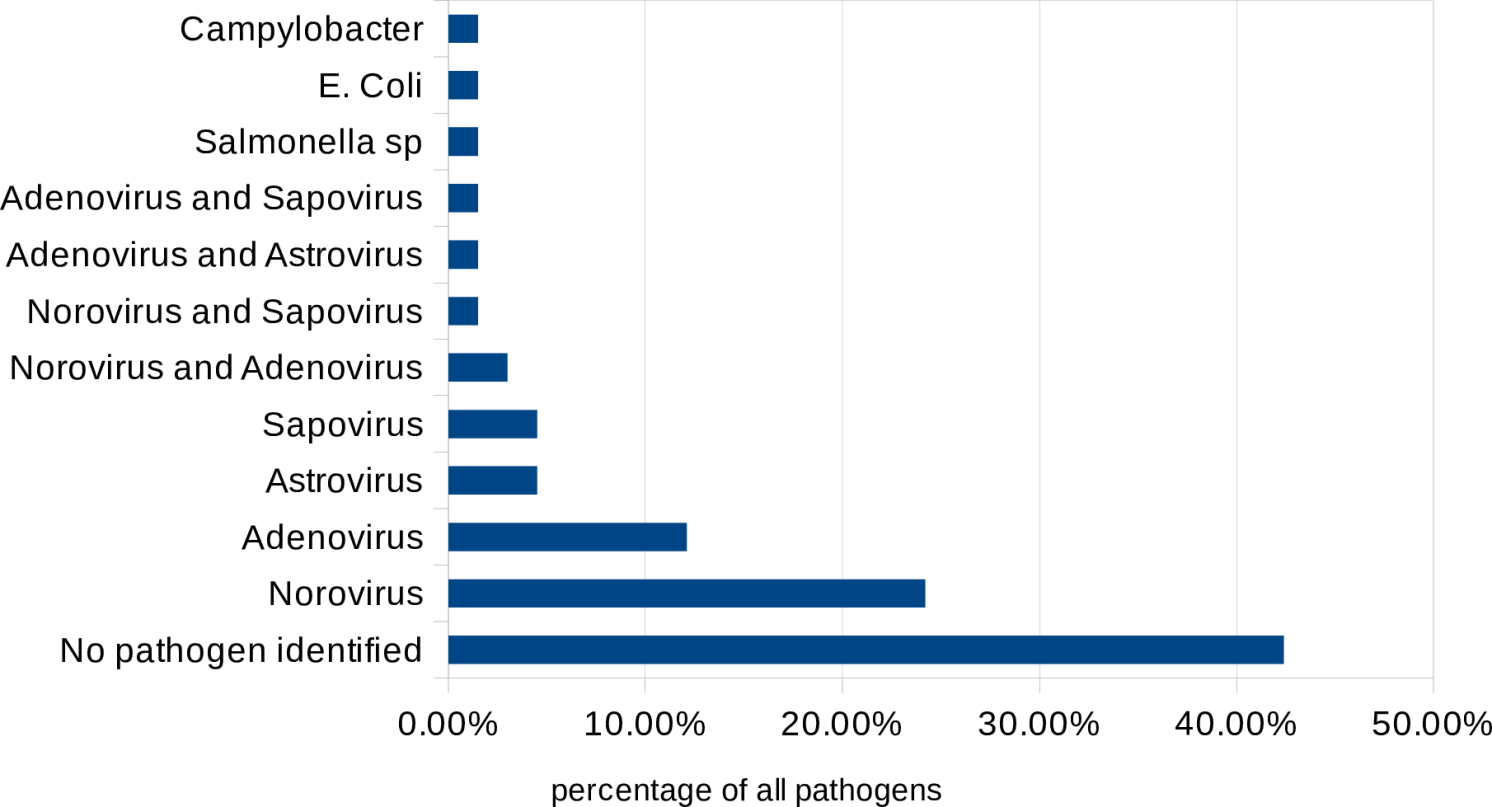
Diarrhoea pathogens identified in the stool sample (RNG group).

**Fig 2:**
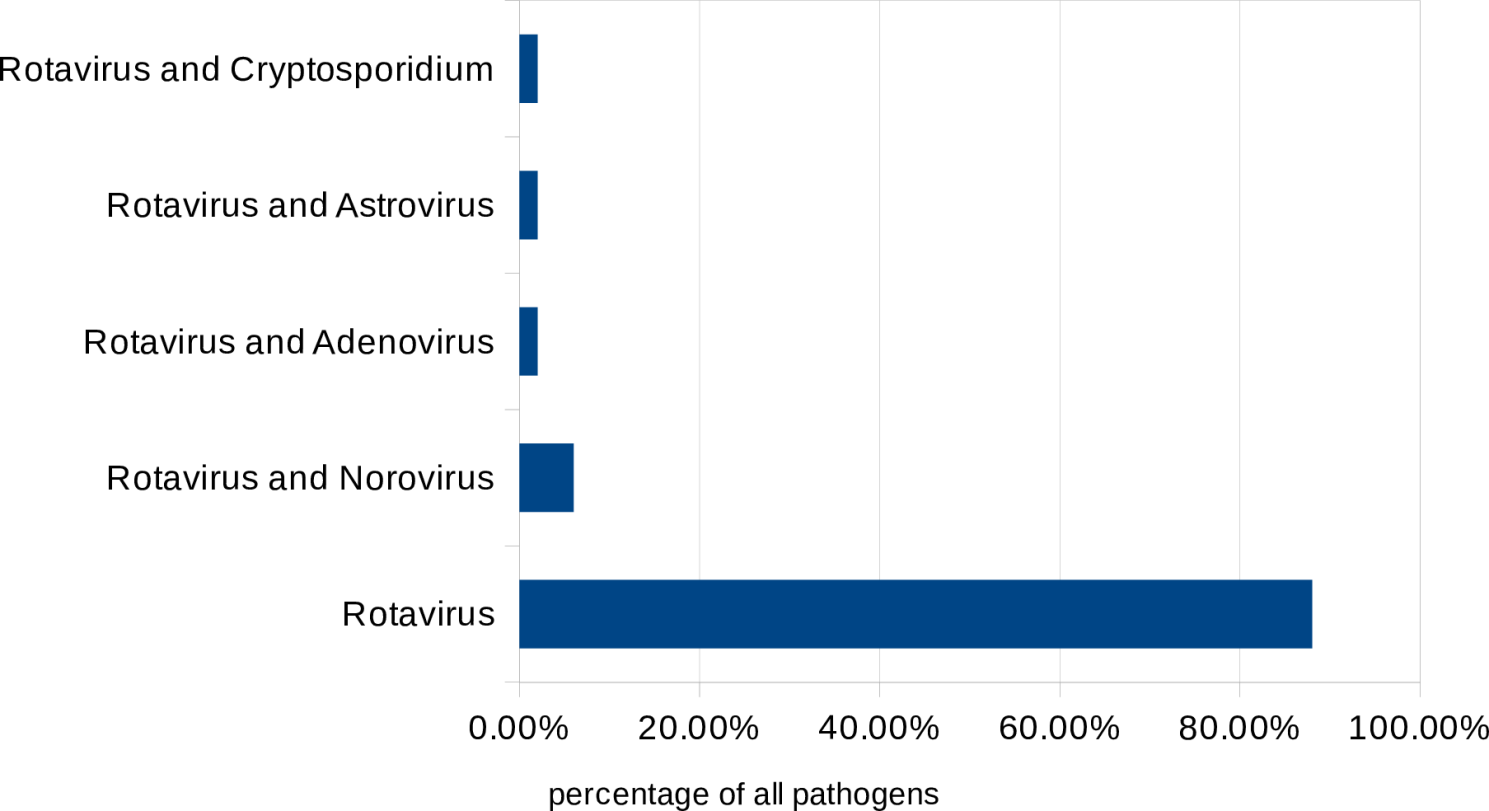
Diarrhoea pathogens identified in the stool sample in addition to rotavirus / Concomitant or secondary GI infection (RPG group).

In the RNG group, norovirus was the most common pathogen (n = 16, 24%), followed by adenovirus (n = 8, 12%), sapovirus (n = 3, 4.5%), astrovirus (n = 3, 4.5%), *Salmonell*a spp. (n = 1, 1.5%), *Campylobacter* spp. (n = 1, 1.5%) and *Escherichia coli* (n = 1, 1.5%); no pathogen was identified in 28 cases (42%); five children (7.5%) were infected with two or more viruses (Fig 1). In the RPG group, six children (12%) had a co-infection with another infectious agent (Fig 2).

### Baseline characteristics and clinical presentation

Baseline characteristics and clinical presentation are shown in Tables 1 and 2, respectively. Children with community-acquired RPG were more likely to be hospitalized than children with community-acquired RNG (93% versus 73%, respectively; P = 0.019). A higher proportion of children with RPG compared to RNG had fever recorded by health care professionals or reported by parents (74% versus 46%; P = 0.005), and children with RPG tended to be more likely to have dehydration (44% versus 26%; P = 0.07) and to get readmitted (6% versus 0%; P = 0.07).

**Table 1:** Demographics of children with rotavirus-positive and rotavirus-negative gastroenteritis.

Variable	Rotavirus Positive Gastroenteritis N = 50	Rotavirus Negative Gastroenteritis N = 66	P value
Age at admission, months; mean (SD)	24 (21)	25 (29)	[matching variable]
Female Sex; n (%)	22 (44%)	33 (50%)	[matching variable]
Community acquired ^a^; n (%)	41 (82%)	60 (91%)	0.17
Underlying chronic medical condition; n (%)	15 (30%)^b^	23 (35%)^c^	0.69
Hospitalized ^d^; n (%) (excluding those with hospital-acquired disease)	38 (93%) [n = 41]	44 (73%) [n = 60]	0.019

SD: Standard Deviation

n shown in square brackets where incomplete data

ª Hospital acquired infection was defined as symptoms that developed 48 hours or more after admission

^b^Pre-existing conditions for rotavirus-positive gastroenteritis: Cardiac (n = 4), Respiratory (n = 6), Gastrointestinal (n = 8), Neurological (n = 5), Haematological (n = 1), Immunodeficiency (n = 1), Renal (n = 1), Chromosomal disorder (n = 3).

^c^ Pre-existing conditions for rotavirus-negative gastroenteritis: Cardiac (n = 1), Respiratory (n = 1), Gastrointestinal (n = 10), Neurological (n = 2), Haematological (n = 3), Immunodeficiency (n = 1), Oncology (n = 3), Malnutrition (n = 3), Chromosomal disorder (n = 1).

^d^ Excluding those with hospital-acquired infection.

**Table 2:** Presentation and management of children with rotavirus-positive and rotavirus-negative gastroenteritis.

Variable	Rotavirus Positive Gastroenteritis N = 50	Rotavirus Negative Gastroenteritis N = 66	P value
Diarrhoea; n (%)	43 (86%)	62 (95%)	0.20
Vomiting; n (%)	37 (74%)	39 (60%)	0.12
Fever; n (%)	37 (74%)	30 (46%)	0.005
Fever, ^o^C (highest); mean (SD)	38.4 (1.0) [n = 46]	37.8 (1.1) [n = 53]	0.009
Total days of illness (from first symptoms to discharge); mean (SD)	4.6 (2.0) [n = 38]	5.3 (4.0) [n = 47]	0.33
Need for Readmission within 28 days; n (%)	3 (6%) [n = 47]	0 (0%)	0.07
Dehydration; n (%)	21 (44%) [n = 48]	16 (26%) [n = 62]	0.07
Received IV fluids; n (%)	25 (50%)	24 (36%)	0.18

SD: Standard Deviation; IV: intravenous

n shown in square brackets where incomplete data

### Laboratory variables

Laboratory variables are shown in Table 3.

**Table 3:** Laboratory results of children with rotavirus-positive and rotavirus-negative gastroenteritis.

Variable	Rotavirus Positive Gastroenteritis N = 50	Rotavirus Negative Gastroenteritis N = 66	P value
pH; mean (SD)	7.30 (0.07) [n = 21]	7.37 (0.06) [n = 11]	0.011
Base excess, mmol/L; mean (SD)	-8.5 (3.7) [n = 21]	-3.9 (3.9) [n = 10]	0.003
Bicarbonate, mmol/L; mean (SD)	17 (3) [n = 21]	21 (3) [n = 10]	0.001
Sodium, mmol/L; mean (SD)	139 (6) [n = 42]	138 (4) [n = 47]	0.87
Potassium, mmol/L; mean (SD)	4.4 (0.6) [n = 39]	4.3 (0.5) [n = 46]	0.40
Creatinine, μmol/L; mean (SD)	39 (14) [n = 42]	34 (13) [n = 47]	0.07
Urea, mmol/L; mean (SD)	5.9 (3.3) [n = 41]	4.2 (1.8) [n = 46]	0.004
Glucose, mmol/L; mean (SD)	4.6 (1.3) [n = 23]	5.2 (1.2) [n = 12]	0.25
ALT, IU/L; mean (SD)	40 (22) [n = 27]	35 (53) [n = 37]	0.007
White cell count, 10^9^ cells/L; mean (SD)	11.4 (7.3) [n = 34]	12.9 (6.7) [n = 45]	0.12
Lymphocytes, 10^9^ cells/L; mean (SD)	2.7 (2.1) [n = 33]	4.4 (3.1) [n = 45]	0.008
Neutrophils, 10^9^ cells/L; mean (SD)	7.5 (6.8) [n = 34]	6.9 (4.9) [n = 45]	0.94
Platelets, 10^9^ cells/L; mean (SD)	299 (117) [n = 34]	317 (111) [n = 45]	0.29
CRP (highest), mg/L; mean (SD)	24 (39) [n = 36]	43 (57) [n = 42]	0.11
Abnormal EEG; n (%)	1 (33%) [n = 3]	0 (0%) [n = 3]	1.00
Abnormal neuroimaging results; n (%)	1 (50%) [n = 2]	0 (0%) [n = 3]	0.40

SD: Standard Deviation; ALT: alanine aminotransferase; CRP: C-reactive protein; EEG: electroencephalogram

n shown in square brackets where incomplete data

Children with RPG compared to RNG had lower blood pH (mean(SD) 7.30(0.07) versus 7.37(0.06), P = 0.011), higher base deficit (8.5(3.7) versus 3.9(3.9) mmol/L; P = 0.003) and lower bicarbonate (17(3) versus 21(3) mmol/L; P = 0.001). There were no significant differences in serum sodium, potassium, glucose, urea, creatinine, alanine aminotransferase, C-reactive protein, neutrophils or lymphocytes between groups.

### Extra-intestinal complications and co-infections

Complications among children with RPG and RNG are shown in Table 4.

**Table 4:** Complications in children with rotavirus-positive and rotavirus-negative gastroenteritis.

Variable	Rotavirus Positive Gastroenteritis N = 50	Rotavirus Negative Gastroenteritis N = 66	P value
Acute GI complications; n (%)	1 (2%)	0 (0%)	0.43
Pathogens from the respiratory tract; n (%)	4 (27%) [n = 15] ^a^	11 (69%) [n = 16] ^b^	0.03
Pathogens from CSF^c^; n (%)	0 (0%) [n = 6]	0 (0%) [n = 5]	1.00
Neurological signs; n (%)	12 (24%)	10 (15%)	0.24
Seizures; n (%)	5 (10%)	8 (12%)	0.78
Reduced conscious level; n (%)	10 (20%)	5 (8%)	0.06
Encephalopathy; n (%)	3 (6%)	0 (0%)	0.08

GI: gastrointestinal; CSF: cerebrospinal fluid

n shown in square brackets where incomplete data

^a^ RPG: Adenovirus (n = 1), Rhinovirus (n = 1), Parainfluenza (n = 1), *Staphylococcus aureus* (n = 1).

^b^ RNG: Adenovirus (n = 4), Rhinovirus (n = 3), Metapneumovirus (n = 2), Enterovirus (n = 2), Respiratory syncytial virus (n = 1), Group G Streptococcus (n = 1).

^c^ Viral studies performed in CSF sample included PCR for herpes simplex viruses 1+2, Varicella Zoster virus, Enterovirus, Cytomegalovirus, Epstein Barr virus. PCR for rotavirus was performed in one sample only.

Neurological complications (seizures, transient reduced consciousness, encephalopathy) were the most common extra-intestinal manifestations of gastroenteritis, but did not differ significantly between children with RPG and RNG (24% versus 15%, respectively; P = 0.24). Cerebrospinal fluid culture and viral PCRs were negative in all cases where a lumbar puncture was performed. In almost all the cases with neurological signs (21/22, 95%), gastroenteritis was community-acquired. Three of the patients had an underlying condition, of whom one had multiple allergies, one iron deficiency anaemia and one hypoxic ischaemic encephalopathy (HIE). There was a trend towards more encephalopathy (3/50 (6%) versus 0/66 (0%), P = 0.08) and reduced consciousness level (10/50 (20%) versus 5/66 (8%), P = 0.06) in children with RPG compared to RNG, respectively. A substantial proportion of children had seizures, but there was no statistically significant difference in prevalence between RPG and RNG groups (10% versus 12%, respectively; P = 0.78). Other stool pathogens identified in association with seizures were norovirus (n = 4, one of whom had co-infection with adenovirus and one with sapovirus) and astrovirus (n = 1). No pathogens were identified in three cases. In a comparison between patients with seizures and/or encephalopathy associated with RPG and patients with RPG who did not have neurological signs, no significant difference was found in dehydration, metabolic acidosis, electrolyte imbalance or hypoglycaemia (S1 Table). A higher proportion of children with RNG compared to RPG were found to have a concomitant pathogen in the upper respiratory tract (69% versus 27%; P = 0.03).

## Discussion

In this case-control study, children with rotavirus gastroenteritis more frequently presented with dehydration, metabolic acidosis and fever compared to children with non-rotavirus gastroenteritis, and were more likely to require hospitalisation. Afebrile and febrile seizures and transient reduced consciousness were noted in a substantial proportion of children from both groups, but encephalopathy was found only in children with rotavirus gastroenteritis.

Dehydration and metabolic acidosis secondary to fluid loss are commonly reported complications of gastroenteritis. The findings of this study are consistent with previous studies globally reporting that rotavirus causes gastroenteritis that is more severe and of longer duration than gastroenteritis caused by other viral pathogens [11,15–18]. One of the striking findings in the present study was the high frequency of neurological complications in previously healthy children presenting with gastroenteritis. Seizures associated with viral gastroenteritis were first described by Morooka in 1982 [19]. The association with rotavirus has been well documented [20,21,22–23]. Other viruses, such as adenovirus, sapovirus and norovirus have also been implicated [24]. The term ‘convulsions with mild gastroenteritis (CwG)’ has been widely used to describe non-febrile seizures that are associated with gastroenteritis in the absence of clinical signs of dehydration or electrolyte imbalance [25–28]. A multicentre study of 128 children from Italy found that the onset of convulsions usually occurs within four days after the onset of gastroenteritis. CwG are generalized in the majority of the cases (87.5%). The duration is usually under five minutes (76%), but prolonged convulsions or status epilepticus may occur [27]. CwG tend to present in clusters [29]. Inter-ictal EEGs are normal in the majority of patients, but even initially abnormal EEGs tend to revert to normal during long-term follow-up [20, 22–24, 27]. The clinical outcome of CwG is good in most children. The possibility of developing epilepsy is low and the use of long-term treatment with antiepileptic drugs is not recommended [22,27]. Rotavirus gastroenteritis is also associated with febrile seizures. Compared to CwG, febrile seizures develop earlier during the course of the illness and tend to last longer [30,31]. However, despite these minor differences, both febrile seizures and CwG are considered to have a good prognosis [28,32].

Our hypothesis was that children with rotavirus gastroenteritis would have higher frequency of seizures and neurological complications than children with non-rotavirus gastroenteritis. Although this was not confirmed, there was a trend towards more encephalopathy, with all 3 cases occurring in the rotavirus-positive group and none in the rotavirus-negative group. Rotavirus has been associated with various encephalopathy syndromes, such as acute necrotising encephalopathy (ANE) [32] and cerebellitis/cerebellopathy [33]. One of the patients in our cohort was diagnosed with MERS, a transient mild encephalopathy with a reversible lesion in the splenium of the corpus callosum on MRI [34]. In patients with MERS, delirious behaviour, disturbance of consciousness or seizures usually present within one week after the onset of symptoms of gastroenteritis and resolve without sequelae within ten days [35,36]. A transient splenial lesion with reduced diffusion that appears as a high signal intensity in diffusion-weighted MRI is the mainstay of diagnosis [37]. Rotavirus is more strongly associated with MERS than any other syndrome of acute encephalopathy in children [38]. In a Japanese national epidemiological survey, rotavirus was the second most common pathogen associated with MERS after influenza virus [38].

The underlying mechamisms of neurological manifestations of rotavirus infection are not completely understood. Detection of rotavirus RNA in CSF of some patients with CNS symptoms supports a hypothesis of direct viral invasion [39]. However, the significance of this finding remains unclear. It has been found that viraemia occurs during the acute phase of disease [40], but most studies in humans have failed to demonstrate a correlation between viraemia and seizures in rotavirus gastroenteritis [41, 42]. Other mechanisms that have been proposed to explain the association between rotavirus infection and seizures focus on the role of the rotavirus non-structural protein 4 (NSP4) [43]. It has been suggested that NSP4 could act either through direct CNS infection (supported by detection of rotavirus RNA in CSF), or indirectly by entering the bloodstream from the intestinal mucosa [43]. NSP4 has been shown to induce nitric oxide metabolites, which are highly reactive free radicals [44]. Nitrites and nitrates are raised in both serum and CSF in patients with rotavirus-associated seizures [45]. It is also known that NSP4 plays a major role in mobilizing intracellular calcium. Rotavirus causes a two- to three-fold increase in intracellular calcium concentration. Disruption of calcium homeostasis induced by NSP4 plays a critical role in the pathogenesis of diarrhoea [46]. It has also been suggested that rotavirus may induce seizures, or increase susceptibility to them, through the same NSP4-induced calcium homeostasis alterations [47]. This hypothesis was tested in a retrospective study of 35 patients in Korea, which found lower serum calcium levels in the patients with seizures compared to those without seizures [47]. However, it remains to be demonstrated whether the reported decreased serum calcium concentration is associated with alterations in neuronal calcium homeostasis.

From a clinical perspective, despite increased use of PCR testing in CSF specimens, the number of cases of childhood encephalitis with unknown aetiology in England is increasing [48], underlining the need for new approaches in the diagnosis of encephalitis. Failure to identify a viral cause could lead to unnecessary antimicrobial treatment. British Guidelines on management of suspected viral encephalitis in children recommend stool PCR testing for enteric viruses as an ancillary investigation in encephalitis associated with gastrointestinal symptoms [49].

An association between rotavirus vaccine and decrease in seizure-related hospitalisation and emergency department visits has been recently suggested. A retrospective analysis of 250,601 infants in the United States showed that full vaccination is associated with an 18–21% reduction in the risk of seizures during the first year after vaccination [50]. A study from Spain reported similar findings with a 16–34% decrease in hospitalisation rates for seizures in children less than 5 years of age following the introduction of rotavirus immunisation [51]. The impact of vaccination was more obvious in infants between 1 and 2 years of age with a 27–52% decrease in hospitalisation rates for seizures. No increase of seizures in older children was found, which might suggest a longer duration for the protective effect of vaccination [51]. It is still unclear whether the protective effect is mainly driven by a reduction in non-febrile or febrile seizures.

This study had strengths and limitations. We were able to select children based on molecular virology results, allowing us to compare clinical features among children with rotavirus compared to other enteropathogens. In the hospital sites in which the study was conducted, blood PCR for enteric viruses is not used for routine diagnostics of acute gastroenteritis, therefore no data on viraemia status were collected. CSF PCR for rotavirus is not part of the routine viral panel for encephalitis and was only requested in one case. We excluded more children than anticipated, predominantly because a fairly high proportion of those with positive PCR results were assessed and treated outside the hospital setting. This led to a relatively small sample size, although cases and controls were well matched on age and sex. The study is limited by its retrospective design, meaning we relied on documentation present in clinical records; inter-observer variation in dehydration assessment and limited use of GCS to access conscious level could have influenced our findings.

## Conclusion

In summary, this study of children presenting with gastroenteritis in three UK hospitals confirmed that rotavirus causes more severe disease compared to other viral pathogens, with children more likely to present with fever, dehydration and metabolic acidosis and require admission or re-admission to hospital. Seizures and milder neurological signs were surprisingly common and associated with multiple pathogens, but encephalopathy occurred only in children with rotavirus-positive gastroenteritis. Therefore, it is important to consider rotavirus infection in the differential diagnosis of a child with encephalopathy or encephalitis, particularly if associated with diarrhoeal disease. Rotavirus vaccination may reduce seizures and presentation to hospital with severe gastroenteritis, but vaccines against other pathogens causing gastroenteritis are required.

## Supporting information

S1 Table Complications in children with rotavirus-positive gastroenteritis with seizures and/or encephalopathy and rotavirus-positive gastroenteritis without neurological symptoms. (ODT)
