# Supplementary material for: Clinical characteristics and complications of rotavirus gastroenteritis in children in east London: A retrospective case-control study
Source: PLoS One. 2018 Mar 22;13(3):e0194009. doi: 10.1371/journal.pone.0194009 (PMC5863974; doi:10.1371/journal.pone.0194009)
Supplement: Supplementary file 1 [file pone.0194009.s001.odt]

S1 Table. Complications in children with rotavirus-positive gastroenteritis with seizures and/or encephalopathy and rotavirus-positive gastroenteritis without neurological symptoms 

| Variable | Rotavirus Positive Gastroenteritis with seizures and/or encephalopathy N=12 | Rotavirus Positive Gastroenteritis without neurological symptoms N=38 | P value |
| --- | --- | --- | --- |
| Age at admission (months); mean (SD) | 26 (17) | 23 (22) | 0.65 |
| Dehydration; n (%) | 2 (29%) [n = 7] | 15 (41%) [n = 38] | 0.69 |
| pH; mean (SD) | 7.27 (0.10) [n = 4] | 7.30 (0.05) [n = 12] | 0.37 |
| Base excess, mmol/L; mean (SD) | -10.45 (4.5) [n = 4] | -8.35 (3.4) [n = 12] | 0.34 |
| Bicarbonate, mmol/L; mean (SD) | 15.3 (2.5) [n = 4] | 17.2 (2.7) [n = 12] | 0.23 |
| Serum sodium, mmol/L; mean (SD) | 138 (5) [n = 6] | 139 (7) [n = 31] | 0.49 |
| Serum creatinine, μmol/L; mean (SD) | 42 (13) [n = 6] | 38 (14) [n = 31] | 0.48 |
| Serum urea, mmol/L; mean (SD) | 6.1 (1.9) [n = 6] | 5.6 (3.4) [n = 31] | 0.71 |
| Glucose, mmol/L; mean (SD) | 4.2 (0.8) [n = 4] | 4.6 (1.3) [n = 14] | 0.56 |
| SD: Standard Deviation # n shown in square brackets where incomplete data |
